# Association between the Hygiene Index Values of Live Fresh Aquatic Products and Food-Borne Diarrhea in the Population of the Ningbo Area in China

**DOI:** 10.3390/ijerph120809154

**Published:** 2015-08-06

**Authors:** Lijun Zhang, Lu Lu, Liye Shu, Jianjun Chen, Baobo Zou, Qi Zhou, Yuanliang Gu, Jinshun Zhao, Xialu Lin

**Affiliations:** 1 Zhejiang Provincial Key Laboratory of Pathological and Physiological Technology, Medical School, Ningbo University, Ningbo 315211, China; E-Mails: zljnb@126.com (L.Z.); zoubaobo@nbu.edu.cn (B.Z.); zhouqi1992@yeah.net (Q.Z.); gurice@yeah.net (Y.G.); 2 Jiangdong Centers for Disease Control and Prevention, Ningbo 315040, China; E-Mails: lulu286559834@163.com (L.L.); hukeli0307@126.com (L.S.); cjj7228554@126.com (J.C.); 3 Key Laboratory of Trace Elements and Endemic Diseases of Ministry of Health, Health Science Center, School of Public Health, Xi’an Jiaotong University, Xi’an 710061, China

**Keywords:** live fresh aquatic products, hygiene index, food-borne diarrhea, association

## Abstract

To investigate the association of the hygiene index values of live fresh aquatic products and food-borne diarrhea in the population of the Ningbo area in China. Volatile basic nitrogen (VBN), histamine (HIS), indole, tetrodotoxin (TTX), and paralytic, neurotoxic, amnesic and diarrhetic shellfish poisons (PSP, NSP, ASP, and DSP, respectively) in the samples of live fresh aquatic products and food-borne diarrhea cases in six studied districts were analyzed. Results indicate that the incidence rate of food-borne diarrhea is related to the hygiene index values. Aside from VBN, the main risk factors related to food-borne diarrhea in edible aquatic products include DSP (in marine fish, shrimp, and other shellfishes), NSP, and ASP (in marine shrimp and crab). Hygiene index values among different species were significantly different. No significant difference in the monitoring index values was found among the six different studied districts. The reported cases of food-borne diarrhea were positively associated with VBN and DSP in aquatic products in Haishu, Jiangbei, Zhenhai, and Beilun, as well as VBN and NSP in aquatic products in Jiangdong and Yinzhou. In conclusion, VBN, DSP, NSP, and ASP are important risk factors for the occurring of food-borne diarrhea in the population of the Ningbo area in China.

## 1. Introduction

Aquatic product poisoning is a significant public health problem in China and around the world [1,2,3,4,5]. Its consumption is an important cause of the food-borne diarrhea. Dai *et al.* reported that, according to the active surveillance of spot hospitals in Jiangsu province in 2008, 14.3% of food-borne illness cases were caused by aquatic product consumption [6]. Li *et al.* reported that seafood ranked the third potential risk food to cause food-borne illness in Guangdong province in 2012 [7]. 18.6% outbreaks of the food-borne diseases could be assigned to the fish category in the United States during 1998–2008 [5]. Epidemiological evidence suggests that the major factors contributing to food-borne diarrhea in aquatic products may depend on their quality, such as freshness, allergen content or biological toxin content [8]. The factors of food-borne diarrhea caused by the aquatic products consumption can be classified into four types: bacterial, parasitic, viral, and chemical causes [9]. The common chemical causes of food-borne diarrhea in aquatic products including histamine (HIS), tetrodotoxin (TTX), paralytic, neurotoxic, amnesic, and diarrhetic shellfish poisons (PSP, NSP, ASP, and DSP, respectively) *etc.* were all established indices and routinely monitored in the Centers for Disease Control and Prevention (CDC) in China [10,11]. PSP could accumulate in the body of *Chlamys farreri* [12], *Mytilus edulis*, oysters [13], *Argopecten irradians*, and *Chlamys Mimachlamys nobilis* [14]. DSP could accumulate in the body of edible shellfish such as scallops, mussels, oysters, and clams [15]. Chen *et al.* reported that, in a food-borne disease outbreak attributed to the consumption of mussels contaminated by DSP, suspension of mussel sales was highly effective in controlling this outbreak [16]. Meanwhile, volatile basic nitrogen (VBN), histamine (HIS), and indole are used as the freshness indicators for the live fresh aquatic products [17]. The US CDC reported that HIS fish poisoning constituted nearly 40% of all seafood-related food-borne illnesses [5].

Ningbo, a city on the eastern sea coast of China, produces a wide variety of aquatic products [18]. Residents in this area are used to eating aquatic products, especially seafood. As a result, seasonal intestinal diseases often break out because of aquatic product consumption [19]. According to the Annual Report (2006) of Ningbo CDC, intestinal diseases related to eating aquatic products accounted for 20.48% of the total number of Notifiable Infectious Diseases. To better monitor the quality of the aquatic products in Ningbo market, live fresh aquatic products were sampled and the amount of VBN, HIS, indole, TTX, PSP, NSP, ASP, and DSP was analyzed. Moreover, an ecological study was performed to investigate the association between the hygiene indices of live fresh aquatic products and the food-borne diarrhea in the Ningbo population.

## 2. Materials and Methods

### 2.1. Sampling

Six out of eleven districts in the Ningbo area were randomly selected in this study, which covered 51.8% of the total household population (481.2 million out of 927.7 million) in 2013. In these six districts, 33 main farm markets and supermarkets were selling live fresh aquatic products, 28 of these were randomly chosen for sample monitoring for this study. Random sampling was conducted on the species having the largest monthly sales. Sampling time was always from the 1st to the 15th of each month. Every month, 100–120 monitoring samples were collected with a total of 1362 samples in a year. Eight categories including 34 species of aquatic products were sampled. They are: freshwater fish category-*Carassius auratus*, *Hypophthalmichthys molitrix*, and *Cyprinus carpio*; marine fish category-*Larimichthys* *polyactis*, *Metynnis hypsauchen*, *Trichiurus lepturus*, *Pneumatophorus japonicus*, *Scomberomorus niphonius*, *Pneumatophorus japonicus*, and *Cynoglossus gracilis*; river crab category-*Eriocheir sinensis*, and *Eriocheir sinensis*; river prawn category-*Macrobranchium nipponense*; sea crab category-*Portunus trituberculatus*, *Lithodes formosae*, and *Portunus pelagicus*; shellfish category-*Ruditapes philippinarum*, *Sinonovacula constrzcta*, *Mactra veneriformis*, *Moerella iridescens*, *Chlamys farreri*, *Neptunea cumingi* Crosse, *Thais clavigera* Kuster, *Babylonia areolata*, and *Corbicula aurea* Heude; shrimp category-*Penaeus* *vannamei* Boone, *Exopalaemon carinicauda*, *Trachypenaeus curvirostris*, *Parapenaeopsis hardwickii*, and *Solenocera* *melantho*; and sleeve-fish category-*Loligo chinensis*, *Sepia officinalis*, and *Octopus vulgaris*.

The data reporting the number of food-borne diarrhea cases was obtained from the China National Epidemic Reporting Network, which was affirmed by the CDC in the six studied districts. To better analyze the correlation between food-borne diarrhea and hygiene indexes, in our study, the food-borne diarrhea cases only included those infectious diarrhea cases caused by aquatic products consumption with epidemiological investigation confirmation. According to *Diagnostic Criteria for Infectious Diarrhea* WS 271-2007 in China, cholera, dysentery, and typhoid were excluded in this study. In addition, hepatitis A, a communicable disease, was also excluded [20].

### 2.2. Monitoring Indices and Methods

The hygiene indices for quality monitoring of live fresh aquatic products were selected according to the most harmful chemicals that potentially cause food-borne diarrhea in the Ningbo area [21], including VBN, HIS, indole, TTX, PSP, NSP, ASP, and DSP. A questionnaire on aquatic products species with the largest monthly sales was completed by the sales managers in the markets in the studied districts [22]. The main contents of the questionnaire included names of the markets, type of sales, species of aquatic products, daily sales, methods of preserving freshness, *etc.* [23]. In addition, the aquatic products were purchased by random sampling with a daily amount (average 1 kg) for a normal three people family, and were packaged in plastic bags and then taken back to the laboratory for immediate analyzing. It is worthy of note that in this field study, no specific permissions were required for these locations/activities because Jiangdong CDC is authorized for the official monitoring the hygiene index values of live fresh aquatic products for the whole Ningbo area. To assure quality control, all samples were pretreated before analyzing which included removing the skin (or shell), eviscerating, gutting, and rinsing with redistilled water (SN/T 1773-2006 and GB/T 5009.212-2008) [24,25]. Then, the edible parts of the samples were selected, chopped, and mixed thoroughly.

VBN was measured by a Semi Micro Kjeldahl Method [26]. A total of 10 g sample was placed in a 250 mL glass conical flask and 100 mL of double-distilled water was added. The mixture was subsequently stirred on a shaker for 30 min, and then filtered with a 0.22 μm filter membrane and 5 mL of the filtrate was added to a Semi Micro Kjeldahl apparatus. After a 5 min steam-distillation, 10 mL of 2% boric acid absorption liquid was added, and then the mixed solution was titrated with hydrogen chloride standard solution. The coefficient of variation (CV) of this method for this sample set was 3.2%.

For the HIS measurement, a 10 g sample was placed in a 50 mL beaker and 30 mL of 10% trichloroacetic acid was added. The mixture was shaken for 3 h, and then filtered with a 0.22 μm filter membrane. The filtrate was extracted with hexane and an azo reagent was added for coloration. The quantity of HIS was detected by colorimetry at a wavelength of 480 nm. The minimum detection level of this method was 5 mg HIS/100 g sample, and the recovery ratio was 97.5% to 102.3%. The two given methods were both in accordance with the CANS L0467 (China National Accreditation Service for Conformity Assessment) and accreditation method GB/T5009.45.

For the indole measurement, a 10 g sample was placed in a 50 mL centrifuge tube and 50 mL methanol and 1 mL 2-methylindole (6.25 μg/mL, an internal standard) were added. The mixture was then homogenized with a high-speed mixing apparatus for 3 min, and then centrifuged for 10 min at 3000 r/min. The supernatant was filtered with a 0.22 μm filter membrane, and 20 μL samples were used for the high-performance liquid chromatography (HPLC) measurement. The mobile phase was methanol:water (60:40); flow rate was 0.8 mL/min; chromatographic column was 30 cm × 4 mm; C18; fluorescence detector excitation wavelength was 280 nm; and the fluorescence wavelength was 330 nm [27]. The indole standard calibration solutions were 6, 12, 24, 36, 48, 60 and 72 μg/L, and the amount of indole was quantified in samples by measuring the height of the indole/2-methylindole peak. The minimum detectable limit of this method was 1 μg indole/100 g sample, CV was 1.5% to 2.8%, and recovery rate was 99.8% to 101.5%, in accordance with AOAC 18.080-51.006.

Indices of toxins were measured with a quantitative enzyme-linked immunosorbent assay (ELISA) kit purchased from Abraxis, the enzyme-labeled meter was MK-3, and results were calculated using a logistic four-parameter model. The minimum detectable limit of TTX was 3.2 ng TTX/100 g samples, CV was 5.2%, and recovery rate was 89.9% to 112.4%. The minimum detectable limit of DSP was 5.6 ng DSP/100 g samples, CV was 4.8%, and recovery rate was 91.39% to 108.20%. The minimum detectable limit of PSP was 1.4 ng PSP/100 g samples, CV was 3.1%, and recovery rate was 77.9% to 115.4%. The minimum detectable limit of ASP was 0.6 ng ASP/100 g sample, CV was 8.9%, and recovery rate was 82.4% to 109.7%. The minimum detectable limit of NSP was 3.3 µg NSP/100 g samples, CV was 4.9%, and recovery rate was 85.4% to 112.3%. The above mentioned quality control for measuring the amount of toxins in each sample was all in the technical parameter range of the kit.

### 2.3. Quality Control

For quality control, five parallel samples were used to measure the VBN in each time sample. A standard recovery was used to determine the accuracy of the results of HIS and indole measurements. An ELISA kit was used to measure the toxins [28]. For the quality control of toxins, five random samples in the same district and month were also analyzed by liquid chromatography-mass spectrometry (LCMS). The coincidence rates of the LCMS and ELISA results were as follows: TTX 90.0% (54/60), PSP 91.7% (55/60), DSP 96.7% (58/60), ASP 86.7% (52/60), NSP 85.0% (51/60).

### 2.4. Statistical Analysis

All measured results were established in an Excel database and analyzed with SPSS 12.0 (SPSS Inc., Chicago, Illinois, USA). One-way ANOVA was used to analyze the differences of hygiene index values in live fresh aquatic products among different months, districts, or different kinds of aquatic products. Pearson correlation analysis was used to analyze the correlation between the number of food-borne diarrhea cases and the hygiene index of live fresh aquatic products. The *p* value less than or equal to 0.05 was considered to have statistical significance. All results are presented as mean ± standard deviation (SD).

## 3. Results

### 3.1. The Time Statistical Distribution Analysis

Table 1 shows the time statistical distribution of different hygiene index values among live fresh aquatic products from January to December. The *F*-values show that, except for indole, all other hygiene index values are significantly different (*p* < 0.05) among different months (January through December). The relatively higher values of VBN were found in July and August, HIS in November, December and January, Indole in March, TTX in January, November and February, PSP in January and February, NSP in June, ASP in January and June, and DSP in August and October, respectively. In addition, although no significant difference of indole values were found among different months, a relative higher amount of indole was detected in some mollusk aquatic products such as squid and octopus.

### 3.2. Location Distribution Analysis

Table 2 shows the location distribution of the different hygiene index values of live fresh aquatic products among six districts. No significant difference in the monitoring index values was found among the six different districts (*p* < 0.05).

### 3.3. Category Distribution Analysis

Table 3 shows the category distribution of the different hygiene index values among different live fresh aquatic products. The hygiene index values were all significantly different among different categories (*p* < 0.05). The mean amount of VBN in shrimp, river prawn, sea crab, and sleeve-fish, indole in shrimp and river prawn, HIS in freshwater and marine fishes, exceeded the related standard limits (see Table 1). Shellfish toxins (PSP, NSP, ASP, and DSP) in the shellfish samples were not found to exceed the related standard limits (see Table 1).

**Table 1:** Time statistical distribution of the different hygiene index values of live fresh aquatic products.

Time	Number of Samples	VBN (mg/100 g) mean ± SD	HIS (mg/100 g) mean ± SD	Indole (mg/100 g) mean ± SD	TTX (μg/kg) mean ± SD	PSP (μg/kg) mean ± SD	NSP (μg/kg) mean ± SD	ASP (ng/kg) mean ± SD	DSP (μg/kg) mean ± SD
January	112	20.58 ± 0.52	193.20 ± 25.87	1.72 ± 0.28	17.32 ± 3.22	3.16 ± 0.39	0.002 ± 0.0004	9.77 ± 1.45	0.43 ± 0.029
February	109	20.64 ± 1.14	46.81 ± 21.78	1.02 ± 0.16	16.03 ± 2.90	3.36 ± 0.42	0.001 ± 0.0003	5.66 ± 0.48	0.40 ± 0.030
March	101	18.73 ± 0.94	22.55 ± 4.36	11.02 ± 10.02	8.86 ± 1.82	1.21 ± 0.19	0.001 ± 0.0003	7.12 ± 0.39	0.40 ± 0.025
April	117	21.28 ± 0.96	26.19 ± 3.23	1.49 ± 0.24	2.71 ± 0.97	1.08 ± 0.24	0.009 ± 0.0017	3.72 ± 0.14	0.39 ± 0.027
May	120	13.97 ± 0.91	61.21 ± 4.96	1.06 ± 0.18	1.85 ± 0.74	0.59 ± 0.07	0.061 ± 0.0055	8.43 ± 1.51	0.50 ± 0.027
June	120	13.57 ± 1.02	63.99 ± 5.08	2.48 ± 0.42	1.15 ± 0.53	0.51 ± 0.06	0.082 ± 0.0080	8.75 ± 2.04	0.37 ± 0.039
July	121	43.45 ± 4.67	87.51 ± 11.53	4.49 ± 1.93	3.04 ± 0.97	1.27 ± 0.22	0.048 ± 0.0071	7.21 ± 0.36	0.51 ± 0.026
August	110	50.15 ± 6.58	65.69 ± 11.90	1.71 ± 0.29	7.59 ± 2.21	0.98 ± 0.18	0.030 ± 0.0020	6.04 ± 0.32	0.81 ± 0.085
September	109	17.31 ± 0.76	160.19 ± 18.92	0.77 ± 0.10	3.18 ± 1.54	0.54 ± 0.09	0.003 ± 0.0006	8.49 ± 1.46	0.66 ± 0.037
October	121	21.28 ± 1.05	113.71 ± 21.30	1.17 ± 0.24	8.23 ± 3.04	0.61 ± 0.17	0.002 ± 0.0005	5.54 ± 0.65	0.79 ± 0.099
November	109	19.94 ± 0.98	309.66 ± 49.79	0.40 ± 0.11	16.33 ± 3.83	1.40 ± 0.47	0.004 ± 0.0014	4.73 ± 0.20	0.49 ± 0.032
December	107	20.72 ± 1.18	253.94 ± 43.78	1.23 ± 0.21	13.26 ± 2.85	2.49 ± 0.38	0.006 ± 0.0014	4.80 ± 0.26	0.50 ± 0.022
*F* **^a^**	—	22.032	11.537	1.072	7.907	12.948	60.839	3.275	7.766
*p*-value ^b^	—	<0.001	<0.001	0.380	<0.001	<0.001	<0.001	<0.001	<0.001
Threshold limit		30, 25, 20, 15, 10 ^c^	100, 30 ^d^	0.05 ^e^	50 ^f^	800 ^g^	800 ^h^	2 × 10^7i^	600, N.D.^j^

Notes: ^a^ *ANOVA* (*n* = 12, α = 0.05). ^b^ *p* < 0.05 was statistically significant. ^c^ VBN: *Hygienic Standard for Fresh and Frozen Marine Products of Animal Origin* in China GB 2733-2005, the standard threshold limit of VBN is 30 mg/100 g in marine fish, shrimp and cephalopod, 25 mg/100 g in sea crab, 20 mg/100 g in freshwater fish and shrimp, 15 mg/100 g in marine shellfish, and 10 mg/100 g in *Gymnocypris przewalskii* and oyster [29]. ^d^ HIS: *Hygienic Standard for Fresh and Frozen Marine Products of Animal Origin* in China GB 2733-2005, the standard threshold limit of HIS is 100 mg/100 g in *Pneumatophorus japonicus*, and 30 mg/100 g in other fishes [30]. ^e^ Indole: U.S. Food and Drug Administration (FDA), the content of indole is used to estimate the freshness of shrimp. For level one freshness, the content of indole should be less than 250 μg/kg; for level three freshness it should be no more than 500 μg/kg (= 0.05 mg/100 g) [31]. ^f^ TTX: The threshold limit of TTX in aquatic product (50 μg/kg) [32]. ^g^ PSP: *Safety Qualification for Agricultral Product-Safety Requirements for Non-environmental Pollution Aquatic Products* GB 18406.4-2001, the standard threshold limit of PSP (800 μg/kg) [33]. ^h^ NSP: U.S. FDA, the standard threshold limit of NSP in clam, mussel, and oyster (800 μg/kg) [34]. ^i^ ASP: *Live and Raw Bivalve Molluscs* CODEX STAN 292-2008, the standard threshold limit of ASP in shellfish (2 × 10**^7^** ng/kg) [35]. ^j^ DSP: *Safety Qualification for Agricultral Product-Safety Requirements for Non-environmental Pollution Aquatic Products* GB 18406.4-2001, the standard threshold limit of DSP (600 μg/kg); *Non-environmental Pollution Food- the Limitation of Harmful Substances in Aquatic Products* NY5073-2006, the standard threshold limit of DSP was “not detectable” (N.D.) in mouse bioassay system, which mean less than 220 μg/kg [36].

**Table 2:** Location distribution of the different hygiene index values of live fresh aquatic products among six districts.

District	Number of Samples	VBN (mg/100 g) mean ± SD	HIS (mg/100 g) mean ± SD	Indole (mg/100 g) mean ± SD	TTX (μg/kg) mean ± SD	PSP (μg/kg) mean ± SD	NSP (μg/kg) mean ± SD	ASP (ng/kg) mean ± SD	DSP (μg/kg) mean ± SD
Haishu	223	20.83 ± 1.09	111.92 ± 16.74	6.39 ± 4.56	8.34 ± 1.82	1.40 ± 0.19	0.023 ± 0.0036	6.39 ± 0.58	0.47 ± 0.024
Jiangdong	221	27.05 ± 2.77	128.38 ± 16.77	1.64 ± 0.22	9.35 ± 1.69	1.85 ± 0.24	0.026 ± 0.0038	7.20 ± 0.87	0.56 ± 0.034
Jiangbei	245	23.79 ± 2.07	109.94 ± 17.25	2.19 ± 0.96	9.70 ± 1.87	1.38 ± 0.19	0.021 ± 0.0033	7.51 ± 1.05	0.53 ± 0.048
Yinzhou	257	25.09 ± 1.76	117.15 ± 18.77	1.22 ± 0.13	8.60 ± 1.57	1.35 ± 0.21	0.016 ± 0.0023	5.83 ± 0.38	0.49 ± 0.020
Zhenhai	197	23.02 ± 1.97	111.56 ± 17.95	1.22 ± 0.16	5.84 ± 1.22	1.25 ± 0.19	0.022 ± 0.0031	7.11 ± 0.73	0.56 ± 0.045
Beilun	213	20.81 ± 1.19	114.22 ± 14.32	1.29 ± 0.15	6.27 ± 1.45	1.23 ± 0.17	0.021 ± 0.0034	6.18 ± 0.53	0.52 ± 0.029
*F* **^a^**	—	1.917	0.181	0.653	0.547	0.935	1.043	1.009	1.360
*p*-value ^b^	—	0.105	0.948	0.621	0.702	0.442	0.384	0.401	0.246

^a^ *ANOVA* (*n* = 6, α = 0.05); ^b^ *p* < 0.05 was statistically significant.

**Table 3:** Category distribution of the different hygiene index values among the different live fresh aquatic products.

Category	Number of Samples	VBN (mg/100 g) mean ± SD	HIS (mg/100 g) mean ± SD	Indole (mg/100 g) mean ± SD	TTX (μg/kg) mean ± SD	PSP (μg/kg) mean ± SD	NSP (μg/kg) mean ± SD	ASP (ng/kg) mean ± SD	DSP (μg/kg) mean ± SD
Shellfish	242	11.21 ± 4.58	43.32 ± 5.98	0.23 ± 0.088	25.04 ± 2.70	3.43 ± 0.253	0.012 ± 0.0013	8.18 ± 0.554	0.49 ± 0.044
Freshwater fish	258	14.45 ± 0.59	117.33 ± 9.09	0.42 ± 0.106	0.14 ± 0.00	0.02 ± 0.009	0.014 ± 0.0019	3.59 ± 0.784	0.26 ± 0.016
Marine fish	376	26.00 ± 1.15	191.13 ± 23.51	2.43 ± 0.186	4.78 ± 0.64	0.88 ± 0.152	0.019 ± 0.0020	6.00 ± 0.573	0.53 ± 0.018
Shrimp	144	34.65 ± 4.58	85.60 ± 14.57	1.37 ± 0.209	4.33 ± 1.46	1.52 ± 0.221	0.035 ± 0.0047	6.66 ± 0.357	0.63 ± 0.042
River prawn	27	22.34 ± 1.08	57.42 ± 4.68	1.14 ± 0.228	0.00 ± 0.00	0.20 ± 0.079	0.010 ± 0.0052	2.00 ± 0.325	0.27 ± 0.044
River crab	22	15.88 ± 1.37	74.56 ± 12.20	0.35 ± 0.132	0.00 ± 0.00	0.05 ± 0.048	0.000 ± 0.0000	1.98 ± 0.261	0.50 ± 0.069
Sea crab	165	34.63 ± 2.73	113.54 ± 13.75	0.93 ± 0.114	12.71 ± 2.00	1.93 ± 0.196	0.040 ± 0.0074	12.09 ± 0.358	0.67 ± 0.055
Sleeve-fish	122	32.71 ± 2.73	79.72 ± 11.05	13.82 ± 6.78	3.19 ± 2.07	1.68 ± 0.288	0.027 ± 0.0034	6.99 ± 0.194	0.83 ± 0.041
*F* **^a^**	—	8.475	9.937	2.298	28.616	36.113	4.838	11.043	11.659
*p*-value ^b^	—	<0.001	<0.001	0.033	<0.001	<0.001	<0.001	<0.001	<0.001

^a^ *ANOVA* (*n* = 8, α = 0.05); ^b^ *p* < 0.05 was statistically significant.

### 3.4. Correlation Analysis

Table 4 shows the reported number of food-borne diarrhea cases during the same time period as the monitoring index values of live fresh aquatic products in the six districts. The food-borne diarrhea cases occurred in July, August, and September accounted for 44.3% (2466/5567) of the total cases for the year. The low-incidence months were April, January, February, and March, respectively.

Table 5 shows the correlation analysis between the reported food-borne diarrhea cases and the monitoring index values of live fresh aquatic products. Food-borne diarrhea case number has a significant correlation (*p* < 0.05) with the amount of VBN or DSP in live fresh aquatic products.

Table 6 shows the correlation between the number of food-borne diarrhea cases and the monitoring index values of live fresh aquatic products among different districts. There was a significant correlation between the number of food-borne diarrhea cases and the amount of VBN (*p* < 0.05) in all six studied districts. A significant correlation between the number of food-borne diarrhea cases and the amount of DSP was also found in most districts (*p* < 0.05). In Yinzhou District, the amount of TTX, PSP or NSP were significantly correlated with the number of food-borne diarrhea cases (*p* < 0.05). In Jiangdong district, the amount of NSP shows a significant correlation with the number of food-borne diarrhea cases (*p* < 0.05).

**Table 4:** Reported case number of the food-borne diarrhea in six districts.

Time	District						Total Case Number
	Haishu	Jiangdong	Jiangbei	Yinzhou	Zhenhai	Beilun	
January	76	27	67	33	11	21	235
February	80	27	62	25	19	15	228
March	70	24	57	31	9	16	207
April	65	18	28	39	16	20	186
May	104	25	52	131	23	90	425
June	118	55	73	186	37	90	559
July	185	63	148	232	49	121	798
August	230	59	152	209	77	244	971
September	173	42	144	195	45	98	697
October	108	34	75	78	34	91	420
November	74	29	81	97	34	96	411
December	118	40	100	98	20	54	430
Total	1401	443	1039	1354	374	956	5567

**Table 5:** The correlation of food-borne diarrhea and the monitoring index of live fresh aquatic products over 12 months.

Correlation	VBN	HIS	Indole	TTX	PSP	NSP	ASP	DSP
Coeff. ^a^	0.686	0.050	−0.166	−0.393	−0.428	0.417	0.153	0.642
*p*-value ^b^	0.007	0.439	0.303	0.103	0.082	0.089	0.318	0.012

^a^ Pearson coefficient (*n* = 12, α = 0.10); *^b^* *p* < 0.05 was statistically significant.

**Table 6:** The correlation of food-borne diarrhea and the monitoring index of live fresh aquatic products in different districts.

District		VBN	HIS	Indole	TTX	PSP	NSP	ASP	DSP
Haishu	Coeff. ^a^	0.743	−0.078	−0.114	−0.390	−0.350	0.330	0.165	0.673
	*p*-value ^b^	0.003	0.405	0.362	0.105	0.132	0.148	0.305	0.008
Jiangdong	Coeff. ^a^	0.644	−0.013	−0.020	−0.346	−0.277	0.532	0.219	0.394
	*p*-value ^b^	0.012	0.485	0.475	0.135	0.191	0.038	0.347	0.103
Jiangbei	Coeff. ^a^	0.686	0.218	−0.084	−0.137	−0.174	0.101	0.152	0.611
	*p*-value ^b^	0.007	0.248	0.397	0.335	0.294	0.377	0.318	0.017
Yinzhou	Coeff. ^a^	0.501	−0.003	−0.140	−0.585	−0.553	0.628	0.288	0.434
	*p*-value ^b^	0.049	0.496	0.333	0.023	0.031	0.014	0.182	0.079
Zhenhai	Coeff. ^a^	0.730	−0.010	−0.239	−0.325	−0.449	0.334	0.001	0.714
	*p*-value ^b^	0.003	0.479	0.227	0.151	0.072	0.145	0.499	0.005
Beilun	Coeff. ^a^	0.708	0.017	−0.220	−0.300	−0.480	0.382	0.019	0.739
	*p*-value ^b^	0.005	0.479	0.247	0.171	0.057	0.109	0.476	0.003

^a^ Pearson coefficient (*n* = 12,α = 0.10); ^b^ *p* < 0.05 was statistically significant.

## 4. Discussion

From January through December in 2013, we sampled the live fresh aquatic products from 28 main markets and supermarkets in six districts in the Ningbo area. The samples selected included 34 species of live fresh aquatic products, which are in high demand and commonly consumed by the Ningbo population. During the same time, food-borne diarrhea cases in these six districts were collected from the epidemiological databases. Thereafter, association between the hygiene index values of live fresh aquatic products and food-borne diarrhea in the population of the Ningbo area was analyzed. Based on the statistical analysis of the dynamic trend of the related hygiene index values, except for that of indole, all other index values were significant different in different seasons. In the first season (January to March), TTX, PSP, and ASP values started from high in January to getting lower until March. The highest value of indole was found in squid in March. Therefore, it is worth noting the influence of high values of shellfish toxins and indole on the occurrence of food-borne diarrhea. VBN remained at a relatively low level during January to March, which might be related with a low temperature (2–14 degrees centigrade) at this time in Ningbo area. From April to June, NSP and ASP tended to range from low to high (NSP: 0.009 μg/kg to 0.082 μg/kg, ASP: 3.72 μg/kg to 8.75 μg/kg), but the other indices remained at a low level during this season (see Table 1). However, during this season, the temperature (12–28 degrees centigrade) is suitable for the growth of aquatic algae and other organisms, especially during water eutrophication, algal toxins such as domoic acid can be produced easily in nitrogen-abundant water [37,38]. These conditions can cause fish, shrimp, crab, or other shellfish to accumulate toxins, and people consuming these products may be exposed to these related toxins. July to September normally represents a hot and humid summer (21–33 degree centigrade, 80%–87% relative humidity) in Ningbo area, as well as the off-season for fishing in China. During this time, most marine products in the markets are freshly preserved by placing them in ice, except for some aquaculture products. Therefore, visceral and nearby tissues might deteriorate, which was proven by the increase of VBN and DSP in this study. The average amount of VBN was 43.45 and 50.15 mg/100 g (75% percentile 34.20 and 37.11 mg/100 g) in July and August, respectively. This analysis suggested that most aquatic products in the markets during these two months belonged to the level 3 in freshness [39]. Residents consuming these aquatic products have an increased risk of food-borne diarrhea, especially for those having a habit of eating uncooked or only short-time steamed food. The main hygienic problems of raw aquatic products included bacterial, virus and parasite infections, and toxins [40]. The epidemiologic study of Zhou suggested that eating medium raw and raw seafood were important risk factors of infectious diarrhea [41]. From October to December, at the end of the off-season fishing, live fresh aquatic products begin to be sold in large quantities in the markets again. During this period, the temperature was low (4–23 degrees centigrade) in the Ningbo area and the amount of VBN in aquatic products also decreased. However, the remarkable variety of deep-sea products coming into the market during this season, such as mackerel, crust deep-sea shrimp, natural reproductive portunid, stone crab, sea crab, snail, and striped snail, which are rare in other seasons, increased, as did the quantity of allergen and shellfish toxins contained in these aquatic products. As the monitoring indices HIS, TTX and PSP started to increase, the number of food-born diarrhea cases in this season was also increased, indicating that unhealthy aquatic products and these biotoxins may play an important role in food-borne diarrhea, especially for those having the neurological and anaphylactic symptoms [16].

In 2009 year, Yuan *et al.* suggested that high VBN values might be positively associated with an increase in food-borne diarrhea cases [42]. Our results indicate that, aside from VBN, DSP (in marine fish, shrimp, crab, river crab, cephalopods, and other shellfish), and NSP and ASP (in marine shrimp and marine crab) are also risk factors for food-borne diarrhea in aquatic products. The number of reported food-borne diarrhea cases was positively associated with VBN and DSP in aquatic products in Haishu, Jiangbei, Zhenhai and Beilun, and also with VBN and NSP in aquatic products in Jiangdong and Yinzhou. According to Zhao *et al.*, the norm of DSP varied from not-detectable to 2000 μg/kg in different standard around the world [36]. The most common method of DSP detection is mouse bioassay, the detection limit of which is 0.05 MU/g (equal to 220 μg/Kg) [43]. The sensitivity of HPLC, LC-MS, and ELISA methods is higher. According to the review of Huang *et al.*, in HPLC method, the detection rate of DSP in shellfish was low (0%–48%) in China [44]. Liu *et al.* reported that the detectable DSP (HPLC) ranged from 0.03 to 7.12 μg/g (30–7120 μg/kg) in 45.3% samples of sea shellfish China coast, 2001 [45]. Xu reported only 2.5% sea shellfish samples exceeded the detection limit of DSP (10 μg/kg, ELISA), and the highest DSP was 95.9 μg/kg in Ningbo area, 2012 [46]. It is in accord with our study; from January to December, the average amount of DSP range from 0.37 to 0.81 μg/kg in Ningbo area, which is also at a very low level. The reported minimal toxic dose of DSP in human was 12 MU (48 μg, 1 MU equal to 4 μg Okadaic acid) [36]. However, the causes of food-borne diarrhea are comprehensive and co-exposure, and combined effects probably exist. The reported DSP is only one of these. The association between DSP and food-borne diarrhea cases in aquatic products need to be further studied.

Indole is generated by the protein tryptophan, metabolized by the bacterial decarboxylase. As sleeve-fish exhibits higher contents of tryptophan, its content of indole can also be used as an index for evaluating the level of freshness. The annual mean content of indole in squid was very high compared with that in the other categories, with the highest value being 1023 mg/100 g in the sample of Haishu district in March. These results remind us that the squid sold in the Ningbo area was not fresh enough. Some reports indicated that the value of indole was positively associated with the degree of deterioration of shrimp [47]. But our results show that the value of indole is not positively associated with VBN in shrimp.

Ningbo is located in the eastern coastal region of China and has a wide variety of aquatic products. Residents in the Ningbo area have a habit of eating aquatic products, especially marine products. It is worth noticing that, in the Ningbo area, most of the time the aquatic products were not cooked or cooked or steamed only for a short time. During the Chinese New Year season, from January to March, residents usually consume more aquatic products. From April to June, before the off-season for fishing, there is a greater supply of aquatic products, and the consumption by residents also remains at a high level due to the relatively low prices. From July to September, although the aquatic products in the markets were not as fresh as other seasons, the prices were low for keeping consumption at a higher level. From October to December, after the fishing off-season, the price increased because of good quality. Therefore, the consumption of aquatic products by residents in Ningbo remained at a high level from January to September, which is in agreement with the occurrence of food-borne diarrhea in the same time period. In summary, we conclude that VBN, DSP, NSP, and ASP in aquatic products are the most important risk factors causing food-borne diarrhea in the Ningbo area from January to September.

As mentioned in the introduction, bacterial, parasitic, and viral factors in the aquatic products may also play important roles during the occurring of the food-borne diarrhea. Therefore, more related risk factors should be taken into consideration for analyzing the association with food-borne diarrhea incidence rates in further studies. Currently, there is no rigorous monitoring program for aquatic products in China [48]. In addition, it is well known that sustained heating couldn’t decrease the toxicity of DSP [15]. Therefore, optimal methods for effectively preventing the occurrence of food-borne diarrhea caused by unsanitary aquatic products are still lacking. Under this situation, public awareness and education might be an alternative method to help prevent and control the food-borne diarrhea in China. Dynamic monitoring of hygienic indices and publishing the results for public may also be helpful in reducing the risk of food-borne diarrhea occurring.

## 5. Conclusions

In present study, we found that VBN, HIS, indole in some categories of live fresh aquatic products in Ningbo markets exceeded the related standard limits, and the hygiene indices varied with the seasons, and VBN, DSP, NSP, and ASP were probable important risk factors for the occurrence of food-borne diarrhea in the population of the Ningbo area in China. And due to the comprehensive causes of food-borne diarrhea, more related risk factors should be taken into consideration and epidemiologic studies with stronger efficacy are needed in further analysis. Public health authorities should raise the awareness of the aquatic product safety.
